# Spontaneous healing and complete disappearance of an intracranial vertebral artery dissecting aneurysm: A case report

**DOI:** 10.1097/MD.0000000000031444

**Published:** 2022-11-25

**Authors:** Qiaowei Wu, Tianxiao Li, Li Li, Kaitao Chang, Qiuji Shao

**Affiliations:** a Cerebrovascular Department of Interventional Center, Zhengzhou University People’s Hospital and Henan Provincial People’s Hospital, Zhengzhou, Henan, China; b Department of Neurosurgery, The First Affiliated Hospital of Harbin Medical University, Harbin, Heilongjiang, China.

**Keywords:** case report, dissecting aneurysm, intracranial aneurysm, spontaneous healing, vertebral artery

## Abstract

Intracranial vertebrobasilar dissecting aneurysms (VBDAs) are associated with a greater tendency to rupture and a greater risk of worse outcomes than anterior circulation aneurysms. Spontaneous healing of a VBDA is very rare, and there have been very few case reports of spontaneous healing of an aneurysm. We describe a case of intracranial vertebral artery dissecting aneurysm that healed spontaneously and disappeared completely on follow-up images.

**Patient concerns:**

A 40-years-old woman was referred to the neurology department because of a persistent headache, especially in the left occiput.

**Diagnoses:**

Magnetic resonance angiography and computed tomography angiography showed a left vertebral artery dissection-like aneurysm (4.5 × 2.0 × 2.5 mm in size) with proximal parent artery mild stenosis (40%).

**Interventions:**

Flunarizine hydrochloride was administered for symptomatic treatment and follow-up angiography was performed.

**Outcomes:**

Digital subtraction angiography and magnetic resonance angiography showed that the aneurysm had completely disappeared at 3 months follow-up. High-resolution magnetic resonance vessel wall imaging revealed intimal thickening and mild stenosis in the left intracranial vertebral artery without an aneurysm signal. In addition, enhancement scanning revealed that the aneurysm area was moderately enhanced. MR-vessel wall imaging at 7 months follow-up showed that the enhancement was slightly reduced compared with the previous time.

**Lessons:**

This case illustrates the relatively plastic nature of a vertebral dissecting aneurysm, indicating that spontaneous healing remains possible.

## 1. Introduction

The incidence of posterior circulation aneurysms is lower than that of other intracranial aneurysms, accounting for approximately 6.6% of all intracranial aneurysms.^[1]^ The Posterior circulation aneurysms, especially vertebrobasilar dissecting aneurysms (VBDAs), are associated with a greater risk of poor outcomes.^[2,3]^ Endovascular therapy including stent-assisted coiling and flow-diversion has certain advantages as compared to open surgical therapies, but posterior circulation aneurysm is still associated with an increased risk of complications after the treatment.^[4,5]^ The majority of neurological complications after treatment were ischemic strokes, followed by hemorrhagic strokes, perianeurysmal edema, vasospasm, and cranial nerve deficit.^[4,5]^ The treatments of these aneurysms are associated with potential risks of morbidity and mortality, and questions arise about whether an unruptured aneurysm should be treated surgically. Spontaneous healing of intracranial aneurysms, especially VBDAs, is very unusual, and there have been very few case reports on the spontaneous healing process. We report the case of a 40-years-old woman with an intracranial vertebral artery dissecting aneurysm that healed spontaneously and disappeared completely on follow-up imaging. The possible etiologies and mechanisms are discussed.

## 2. Case presentation

**Figure 1.**
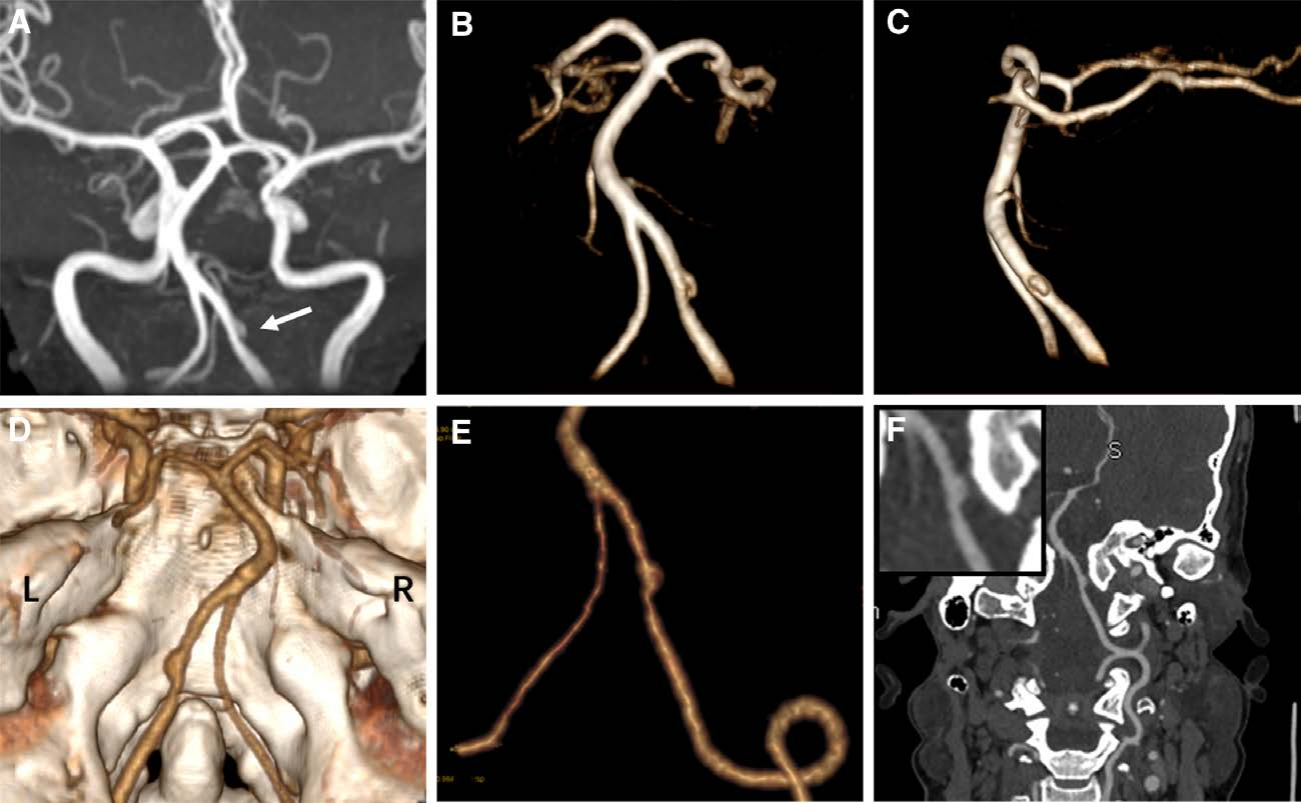
Maximum intensity projection (MIP) time-of-flight magnetic resonance angiography (TOF MRA) (A), anterior (B), and lateral projection (C) of 3-dimensional TOF MRA reconstruction image, 3-dimensional reconstruction of computed tomography angiography (CTA) (D, E), and MIP image (F) revealed a left vertebral artery dissection-like aneurysm (arrow) (4.5 × 2.0 × 2.5 mm in size) with mild stenosis of the proximal parent artery (40%). CTA = computed tomography angiography, MIP = maximum intensity projection, TOF MRA = time-of-flight magnetic resonance angiography.

**Figure 2.**
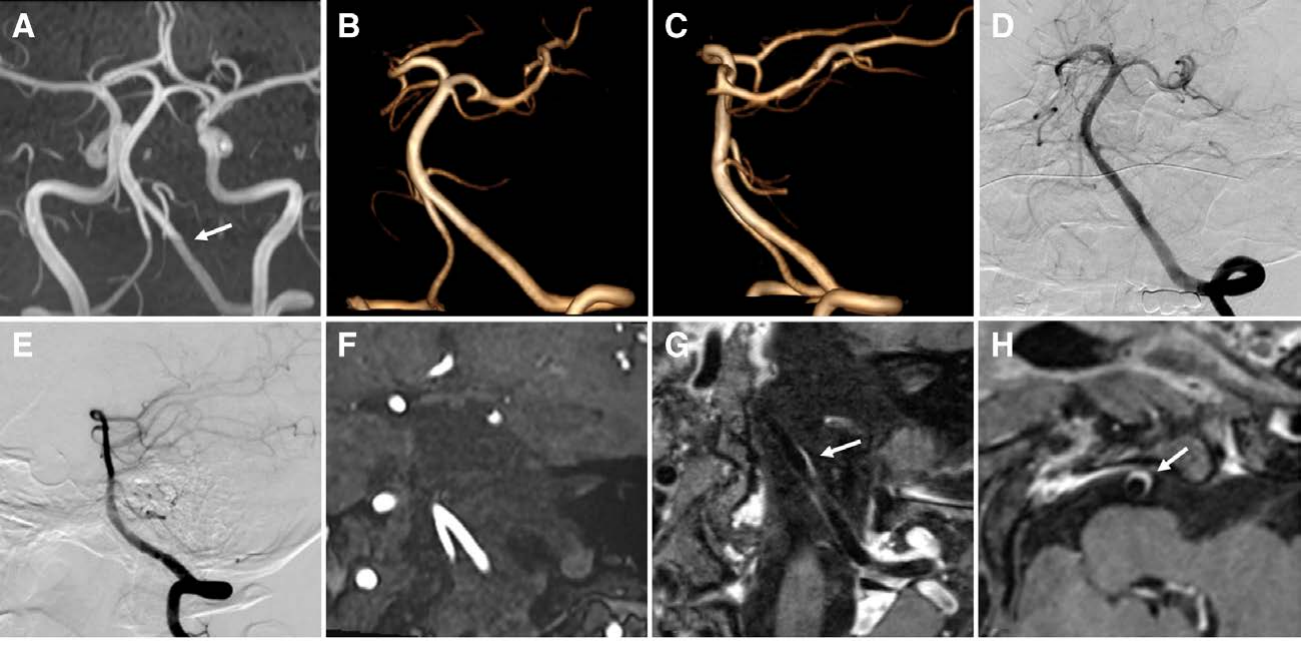
Maximum intensity projection (MIP) time-of-flight magnetic resonance angiography (TOF MRA) (A), anterior (B) and lateral projection (C) of 3-dimensional TOF MRA reconstruction image, digital subtraction angiography (D, E), and source image of TOF sequence (F) at 3 months follow-up showed the aneurysm was spontaneously healed and completely disappeared. The high-resolution magnetic resonance vessel wall imaging showed the intimal thickening and mild stenosis in the left intracranial vertebral artery, without aneurysm signal (G). In addition, the enhancement scanning showed that the former aneurysm area was moderate enhanced (G, H) (arrow). MIP = maximum intensity projection, TOF MRA = time-of-flight magnetic resonance angiography.

**Figure 3.**
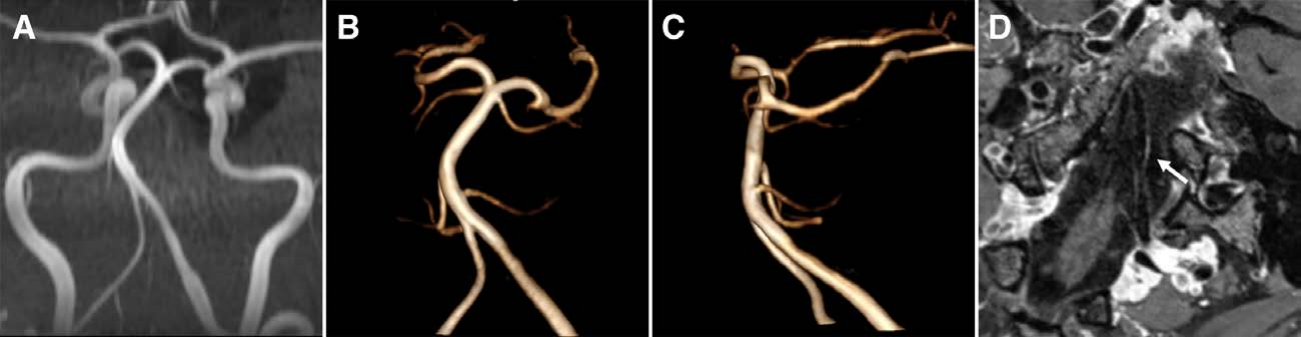
Maximum intensity projection (MIP) time-of-flight magnetic resonance angiography (TOF MRA) (A), anterior (B) and lateral projection (C) of 3-dimensional TOF MRA reconstruction image at 7 months follow-up showed the persistence disappearing of the aneurysm, and the enhancement was slightly reduced as compared to the last time (D) (arrow). MIP = maximum intensity projection, TOF MRA = time-of-flight magnetic resonance angiography.

Written informed consent was obtained from the patients, and the study was approved by the institutional review board of Henan Provincial People’s Hospital. A 40-years-old woman visited our clinic because of persistent, tolerable headaches for one day, especially in the left occiput. The patient denied any medical or traumatic history, family history of intracranial aneurysms, history of oral contraceptive use, or history of hypertension or diabetes mellitus. A physical examination revealed no abnormalities. Subsequently magnetic resonance imaging showed no brain parenchyma abnormalities, while magnetic resonance angiography showed a left vertebral artery dissection-like aneurysm (4.5 *×* 2.0 *×* 2.5 mm in size) with proximal parent artery mild stenosis (40%). No intracranial hemorrhage or subarachnoid hemorrhage (SAH) was observed on cranial computed tomography. Computed tomography angiography also revealed a left vertebral artery aneurysm (Fig. 1). After the administration of flunarizine hydrochloride for symptomatic treatment, the clinical symptoms improved, and the patient refused further surgical treatment. Therefore, careful observation was made. The patient did not show any clinical symptoms at 3 months follow-up. Digital subtraction angiography and magnetic resonance angiography showed that the aneurysm had completely disappeared. High-resolution magnetic resonance (HRMR) vessel wall imaging (VWI) revealed intimal thickening and mild stenosis in the left intracranial vertebral artery without an aneurysm signal. In addition, enhancement scanning revealed that the aneurysm area was moderately enhanced (Fig. 2). Laboratory tests, including routine blood tests, C-reactive protein, erythrocyte sedimentation rate, anti-dsDNA antibody, anti-nuclear antibodies, extractable nuclear antigens, and anti-cyclic citrullinated peptide antibodies, did not reveal any abnormalities. MR-VWI at 7 months follow-up showed persistent disappearance of the aneurysm, and the enhancement was slightly reduced compared to the previous time (Fig. 3). The timeline of the follow-up imaging results is presented in Table 1.

**Table 1:** The timeline of imaging follow-up results.

Imaging methods	Baseline images	3 months	7 months
CTA	A left VADA	–	–
MRA	A left VADA	No aneurysm was detected	No aneurysm was detected
HRMR-VWI	–	No aneurysm was detected Intimal thickening Moderately enhanced	No aneurysm was detected Slightly enhanced
DSA	–	No aneurysm was detected	–

CTA = computed tomography angiography, DSA = digital subtraction angiography, HRMR-VWI = high-resolution magnetic resonance vessel wall imaging, MRA = magnetic resonance angiography, VADA = vertebral artery dissecting aneurysm.

## 3. Discussion

Intracranial VBDAs are recognized as potential causes of SAH or posterior circulation ischemic stroke in young and middle-aged adults.^[6]^ The natural history of posterior circulation aneurysms is generally thought to be highly dangerous. Published reports have shown that posterior circulation aneurysms have a greater tendency to rupture than anterior circulation aneurysms do.^[7,8]^ Nasr et al^[9]^ reported a poor natural prognosis of VBDAs with growth rates of 6.5%/year and rupture rates of 1.5%/year. Once ruptured, subsequent re-rupture occurred in more than 70% of the patients, and over 50% of the re-ruptures occurred within 24 hours after the first SAH, resulting in a mortality rate of 46.7%.^[10]^

The formation of intracranial VBDA is associated with multiple intrinsic and environmental factors, such as congenitality, drug usage (e.g., oral contraceptives), infection, systemic lupus erythematosus, hypertension, diabetes mellitus, history of migraine, polycystic kidney disease, syphilis, polyarteritis nodosa, Moyamoya disease, or traumatic origin, which could result in the circulation of blood diverting into the weakened vessel wall because of sudden disruption of both the internal elastic lamina and media.^[11–13]^ In our case, the relationship between recurrent localized headache and intracranial vertebral artery dissecting aneurysm was elusive; however, reports have suggested that vessel wall edema during migraine may be associated with the development of intracranial artery dissection.^[14]^ No definite evidence of infection, other physical disorders, history of injury, or congenital disorders was found in this case.

In addition to several case reports, there are limited data on the spontaneous healing of intracranial aneurysms in adults.^[15–19]^ Most reported aneurysms are located in small perforator arteries or distal arteries,^[16–19]^ except for a tiny (1 mm) blood blister-like aneurysm, which is located in the internal carotid artery.^[15]^ No Vertebrobasilar artery trunk aneurysms have not been reported.

In our case, we first reported the spontaneous healing and complete disappearance of an intracranial vertebral artery dissecting aneurysm. The mechanisms and factors influencing the spontaneous healing of aneurysms have not been elucidated. Spontaneous intra-aneurysmal thrombosis is a possible cause.^[20,21]^ The intra-aneurysmal thrombus could reduce or, in extreme cases, completely occlude the aneurysm and cease the further dissection of the parent artery, leading to the complete occlusion and disappearance of the aneurysm.

The self-repair of the vessel wall may also be a potential mechanism of action. In dissecting aneurysms with spontaneous healing, cessation of blood flow into the aneurysmal sac may initiate the healing process. In this case, VWI showed that the former aneurysm area was enhanced. Shimonaga et al^[22]^ reported 9 intracranial aneurysms that were imaged with enhanced VWI and subsequently subjected to histopathological analyses. The results showed that vessel wall enhancement was associated with neovascularization and macrophage-infiltrated inflammation, which could imply vessel wall remodeling. In addition, vessel wall enhancement reflects intimal hyperplasia and replacement of the hematoma within the aneurysm by granulation tissue during the healing process, which may play a role in enhancing disruption defects and normalizing local hemodynamics.^[23,24]^ However, the lacking of the baseline HRMR-VWI is a limitation to our study. HRMR-VWI has certain advantages in visualizing the vessel or aneurysm wall structures and intra-aneurysmal thrombus, and the wall enhancement provides the mural inflammation information, which could reflect the dynamic changes of vessel and aneurysm wall.^[22,25]^ The use of HRMR-VWI may be an optional modality for the follow-up surveillance of intracranial aneurysms.

## 4. Conclusion

In summary, we present a case of intracranial vertebral artery dissecting aneurysm that spontaneously healed and completely disappeared on follow-up angiography. Although the exact mechanisms and influencing factors of spontaneous healing of aneurysms have not been elucidated, our case illustrates the relatively plastic nature of a vertebral dissecting aneurysm, indicating that spontaneous healing remains a possibility. For asymptomatic patients who are unwilling to undergo endovascular treatment or have aneurysms with a rupture risk that does not exceed procedure-related morbidity, conservative management with close imaging monitoring could be an alternative choice. The pathological and hemodynamic mechanisms should be further investigated to gain deeper insights into the spontaneous healing of intracranial aneurysms.

## Author contributions

**Conceptualization:** Qiaowei Wu, Qiuji Shao.

**Data curation:** Qiaowei Wu, Tianxiao Li, Li Li, Kaitao Chang, Qiuji Shao.

**Formal analysis:** Kaitao Chang.

**Funding acquisition:** Qiuji Shao.

**Investigation:** Qiaowei Wu, Li Li, Qiuji Shao.

**Methodology:** Qiaowei Wu, Tianxiao Li, Li Li, Qiuji Shao.

**Project administration:** Qiaowei Wu, Tianxiao Li, Li Li, Kaitao Chang, Qiuji Shao.

**Resources:** Qiaowei Wu, Qiuji Shao.

**Supervision:** Tianxiao Li, Qiuji Shao.

**Validation:** Tianxiao Li, Qiuji Shao.

**Visualization:** Tianxiao Li, Qiuji Shao.

**Writing ‐ original draft:** Qiaowei Wu, Qiuji Shao.

**Writing – review & editing:** Qiaowei Wu, Qiuji Shao.
